# Inhibition of Apoplastic Calmodulin Impairs Calcium Homeostasis and Cell Wall Modeling during *Cedrus deodara* Pollen Tube Growth

**DOI:** 10.1371/journal.pone.0055411

**Published:** 2013-02-06

**Authors:** Li Wang, Xueqin Lv, Hong Li, Min Zhang, Hong Wang, Biao Jin, Tong Chen

**Affiliations:** 1 Key Laboratory of Plant Molecular Physiology, Institute of Botany, Chinese Academy of Sciences, Beijing, China; 2 College of Horticulture and Plant Protection, Yangzhou University, Yangzhou, Jiangsu, China; 3 College of Biological Science and Technology, Yangzhou University, Yangzhou, China; Iowa State University, United States of America

## Abstract

Calmodulin (CaM) is one of the most well-studied Ca^2+^ transducers in eukaryotic cells. It is known to regulate the activity of numerous proteins with diverse cellular functions; however, the functions of apoplastic CaM in plant cells are still poorly understood. By combining pharmacological analysis and microscopic techniques, we investigated the involvement of apoplastic CaM in pollen tube growth of *Cedrus deodara* (Roxb.) Loud. It was found that the tip-focused calcium gradient was rapidly disturbed as one of the early events after application of pharmacological agents, while the cytoplasmic organization was not significantly affected. The deposition and distribution of acidic pectins and esterified pectins were also dramatically changed, further perturbing the normal modeling of the cell wall. Several protein candidates from different functional categories may be involved in the responses to inhibition of apoplastic CaM. These results revealed that apoplastic CaM functions to maintain the tip-focused calcium gradient and to modulate the distribution/transformation of pectins during pollen tube growth.

## Introduction

Pollen tubes are polarized growing cells capable of orienting themselves inside female tissue to fertilize the ovule. They exhibit a tip-to-base cytoplasmic calcium gradient that is mainly established by extracellular calcium influx, which plays a key role in polarity establishment and maintenance in tip-growing cells [1], [2]. Specific molecular decoders such as calmodulin (CaM) are essential for sensing, interpreting, and transducing of the characteristic Ca^2+^ signature.

CaM has been extensively investigated in both plant and animal cells. It is implicated in regulating a variety of cellular functions and physiological processes, including DNA synthesis and cell division [3], [4], phytochrome-mediated gene expression and chloroplast development [5], gravitropism [6], [7], and microtubule organization [8]. Moreover, it has been documented that CaM may be also located extracellularly and, therefore, may have substantial functions outside cells [9]. The presence of apoplastic CaM was first reported in soluble extracts of oat coleoptile cell wall preparations as determined by radioimmunoassay [10]. Subsequently, there has been further evidence for the existence and putative functions of CaM in the extracellular spaces of different plant cells [11], [12], [13].

There have been some studies on the functions of apoplastic CaM on pollen germination and tube growth [14], but most of them have focused on collecting physiological data for the germination rate and tube elongation in angiosperm species [12], [15], and only a few have reported data on down-stream cytological events. In contrast to angiosperm species, pollen tubes of coniferous species are characterized by an extended period of growth, extremely delayed gametogenesis, special characteristics of cell wall modeling, and control of cytoskeletal components [16]. These differences represent major an evolutionary divergence in the development of male gametophytes in flowering plants [16], [17], [18]. Therefore, it is of great interest to dissect the cytological changes in response to disturbances or blockages in signalling, particularly in the tip-focused calcium gradient, distribution and configuration of cell wall components, and protein expression profiles.

The present study was carried out to examine the cellular responses to inhibition of apoplastic CaM in pollen tubes of *Cedrus deodara* (Roxb.) Loud. Two cell-impermeable antagonists of apoplastic CaM were used–anti-CaM and W7-agarose–and particular attention was paid to their effects on intracellular calcium homeostasis and cell wall modeling. These data may provide new insights into the modulation of apoplastic CaM signalling and the evolutionary divergence of gymnosperm pollen tubes in terms of their tip growth machinery.

## Results

### Anti-calmodulin and W7-agarose Significantly Inhibited Pollen Germination and Tube Growth

**Figure 1:**
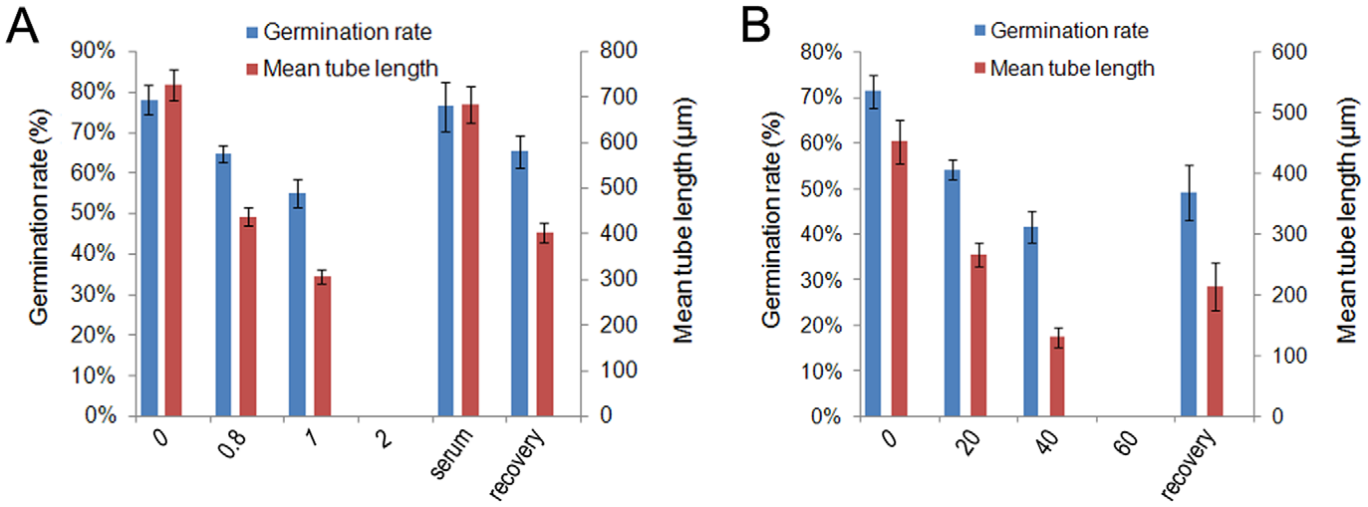
Inhibitory effects of anti-CaM and W7-agarose on pollen germination and pollen tube growth. A, Inhibitory effect of anti-CaM on pollen germination and tube elongation. Numbers on X-axis indicate concentrations of anti-CaM. Pollen tubes incubated in the presence of 1 µg/mL anti-CaM were collected to remove the pharmacological agent, then pollen tubes were further incubated in standard medium for recovery tests before statistical analysis. B, Inhibitory effect of W7-agarose on pollen germination and tube elongation. Numbers on X-axis indicate concentrations of W7-agarose. Pollen tubes incubated in the presence of 40 µM W7-agarose were collected to remove the pharmacological agent, then pollen tubes were further incubated in standard medium for recovery tests before statistical analysis.

The anti-calmodulin antibody (Anti-CaM) drastically inhibited *C. deodara* pollen germination and tube growth in a dose-dependent manner (Figure 1A). Microscopic examinations indicated high viability of pollen in the standard medium with a germination rate of approximately 75% after 54 h of incubation, while 0.8 and 1.0 µg/mL anti-CaM treatments significantly decreased the germination rates to 64% and 55% of that of the control cells, respectively. When the concentration of anti-CaM was increased to 2.0 µg/mL, pollen germination ceased, while the same amount of mouse serum had no significant effect (Figure 1A). After treatments with anti-CaM, pollen tube elongation was also markedly inhibited (Figure 1A). The mean growth rate of pollen tubes was 3.75 µm/h and 2.58 µm/h after treatments with 0.8 µg/mL and 1.0 µg/mL anti-CaM, respectively, whereas it was 5.67 µm/h in the control after 120 h of incubation. Few morphological abnormalities were observed in the anti-CaM treatment. Treatment with 1.0 µg/mL monoclonal anti-green fluorescent protein antibody did not significantly affect pollen germination and tube elongation, and exogenous CaM partly recovered the inhibitory effects of anti-CaM on pollen germination and tube elongation (Figure S1).

**Figure 2:**
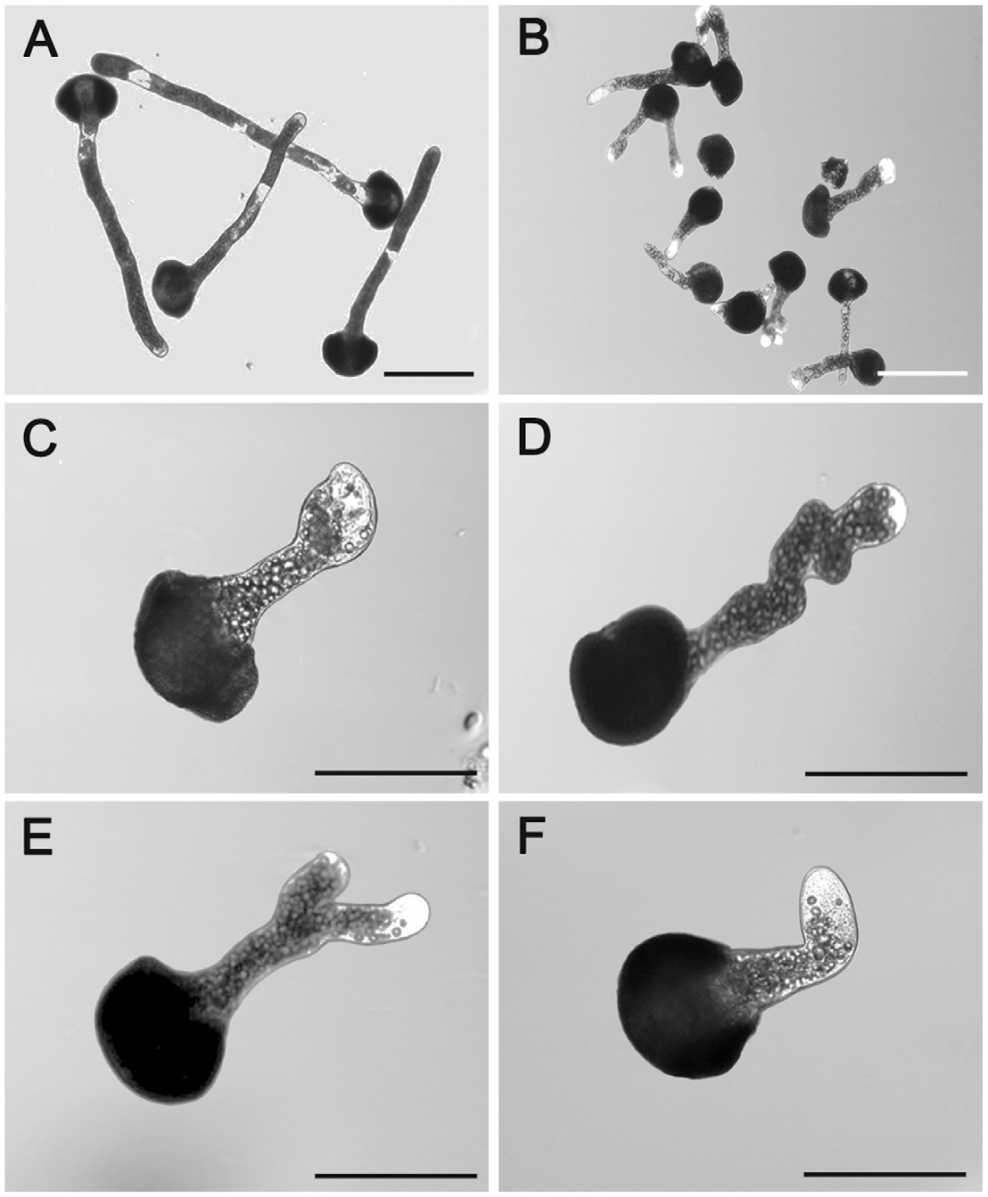
Abnormal morphology of *Cedrus deodara* pollen tubes induced by 20 µM W7-agarose treatment. A, Untreated pollen tubes incubated in standard medium. B–F, Pollen tubes incubated with 20 µM W7-agarose. C, Tube with swollen tip. D: Twisted tube. E: Branched tube. F: Bent tube. A–B: Bar = 200 µm; C–F: Bar = 100 µm.

W7-agarose also inhibited pollen germination and tube elongation in a similar pattern (Figure 1B). In contrast to the anti-CaM treatment, W7-agarose dramatically reduced pollen germination and tube elongation, and a small percentage of severe morphological abnormalities, such as tip swelling, tube branching and bursting, were observed (Figure 2C–F), especially in response to higher concentrations of the inhibitor. Pollen tubes incubated in the standard medium were healthy with normal diameter, length, and shape (Figure 2A). The effects of W7-agarose may be attributed to its naphthalene ring, which is the largest structure that can fit into the hydrophobic binding pockets of Ca^2+^-loaded CaM. Pollen tube development was completely blocked by 60 µM W7-agarose. To ensure that there was no free W7 in the preparation, the W7-agarose beads were extensively rinsed with the culture solution, and the effects of the washed beads and their washing fluid on pollen germination and tube elongation were evaluated. Exogenous CaM (10^−9^ and 10^−8^ mol/L) was also included in the test to outcompete W7 agarose. The results showed that the washing solution did not significantly affect pollen germination and tube elongation, but the rinsed agarose-beads retained some inhibitory effects (Figure S2).

### Dysfunction of Apoplastic CaM Rapidly Disturbed Cytoplasmic [Ca^2+^]_c_ Gradient

**Figure 3:**
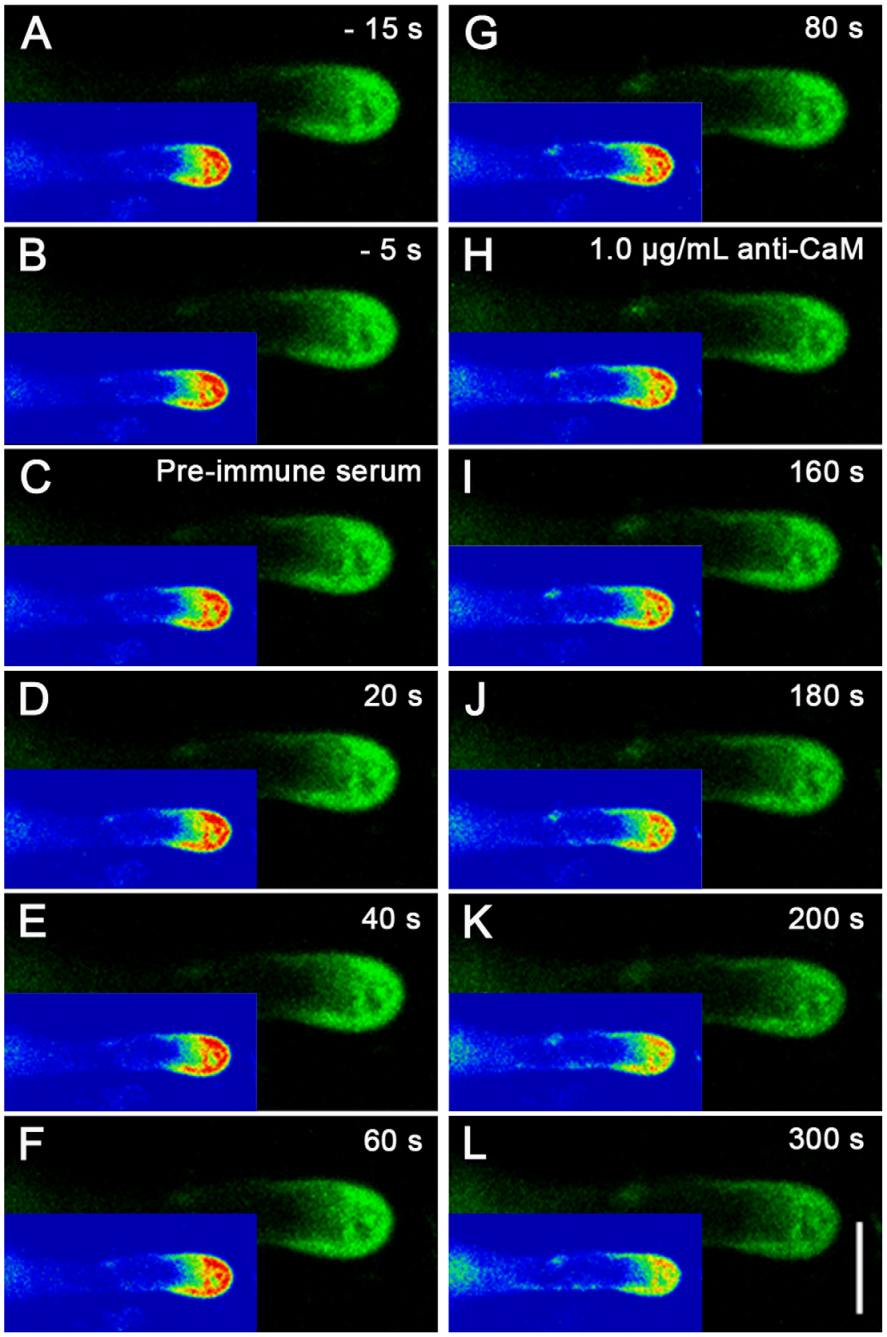
Time course analysis of [Ca^2+^]_c_ changes upon anti-CaM treatment. Calcium Green-1 Dextran microinjection was carried out to further elucidate changes in calcium gradient upon addition of anti-CaM. Calcium Green-1 dextran (2.5 mM in 5 mM HEPES buffer, pH 7.0) was pressure-injected into pollen tubes. Ca^2+^ dynamics in injected pollen tubes were recorded using a LSM 510 META LSCM (Zeiss Co., Germany) in time-course mode (488 nm excitation laser and 525/550 nm band pass emission filter). A–B, Before time-series recording, two single images were captured at 10-s interval to examine fluorescence. C–G, Ca^2+^ distribution in injected pollen tubes did not change significantly after application of pre-immune serum at 0 s. H–L, Ca^2+^ gradient dissipated after addition of 1.0 µg/mL anti-CaM at 100 s during time-course recording. Corresponding rainbow mode images are shown in bottom left corner of each image panel. Bar = 20 µm. Injection, image collection, and subsequent data analysis was carried out for five to seven pollen tubes.

There was a steep gradient of [Ca^2+^]_c_ from the tip to the base of the pollen tubes. The ratio of the fluorescence of cytoplasmic [Ca^2+^]_c_ at the extreme apex to that at the base of the clear zone could be used to quantify fluctuations in the tip-focused [Ca^2+^]_c_ gradient. The pollen tubes cultured in the standard medium showed an almost 3-fold tip-focused gradient (a tip-to-base ratio of 2.57±0.19, n = 9) (Figure S3), which was comparable to the 2-fold tip-focused gradient reported for Norway spruce (*Picea abies*) [17]. This typical tip-focused [Ca^2+^]_c_ appeared relatively constant during the first 300 s and did not change significantly after application of mouse pre-immune serum for 80 s. In contrast, the gradient became significantly shallower over time in the extreme apex after application of 1.0 µg/mL anti-CaM, although the gradient was still detectable. After approximately 300 s, the [Ca^2+^]_c_ gradient severely dissipated and only a weak gradient was observed in the extreme tip (Figure 3). In contrast, pollen tubes cultured in medium containing 1.0 µg/mL anti-CaM displayed a significantly shallower calcium gradient (1.55±0.05, *n* = 7), which was similar to the results obtained from labeling with membrane-permeable Fluo-3/AM ester (Figure S4). Similarly, W7-agarose induced a significant decrease in cytoplasmic [Ca^2+^]_c_ (Figure S5)_._

To further confirm the changes in [Ca^2+^]_c_ as one of the early events upon inhibition on apoplastic CaM, we measured net Ca^2+^ flux at the extreme apex of growing pollen tubes using the scanning ion-selective electrode technique (Figure S6). The results showed the prevalence of Ca^2+^ influx in the control tube apex. The mean maximal Ca^2+^ influx at the peak of the oscillations was 70.83±17.63 pmol cm^−2^ s^−1^ (*n* = 5; Figure S6A). The magnitude of the Ca^2+^ influx at the extreme apex was markedly increased, and then it fluctuated between influx and efflux (48.05±16.22 pmol cm^−2^ s^−1^; *n* = 5) after 300 s of 1.5 µg/mL anti-CaM treatment, indicating that the cytoplasmic [Ca^2+^]_c_ derived from extracellular Ca^2+^ bulk substantially decreased. Given that it may take some time for the pharmacological agents to diffuse in the test solution system, the decreases in net calcium flux can be detected within about 150 s (Figure S6A), implying that the immediate decrease in net calcium influx may be one of the early events triggering the decrease in the tip-focused calcium gradient. W7-agrose also induced similar changes in Ca^2+^ influx (a decrease from 79.64±34.22 pmol cm^−2^ s^−1^ (*n* = 5) to 24.56±39.76 pmol cm^−2^ s^−1^ (*n* = 5, Figure S6B).

### Ultrastructural Analysis of Tip Region after Application of Apoplastic CaM Antagonists

**Figure 4:**
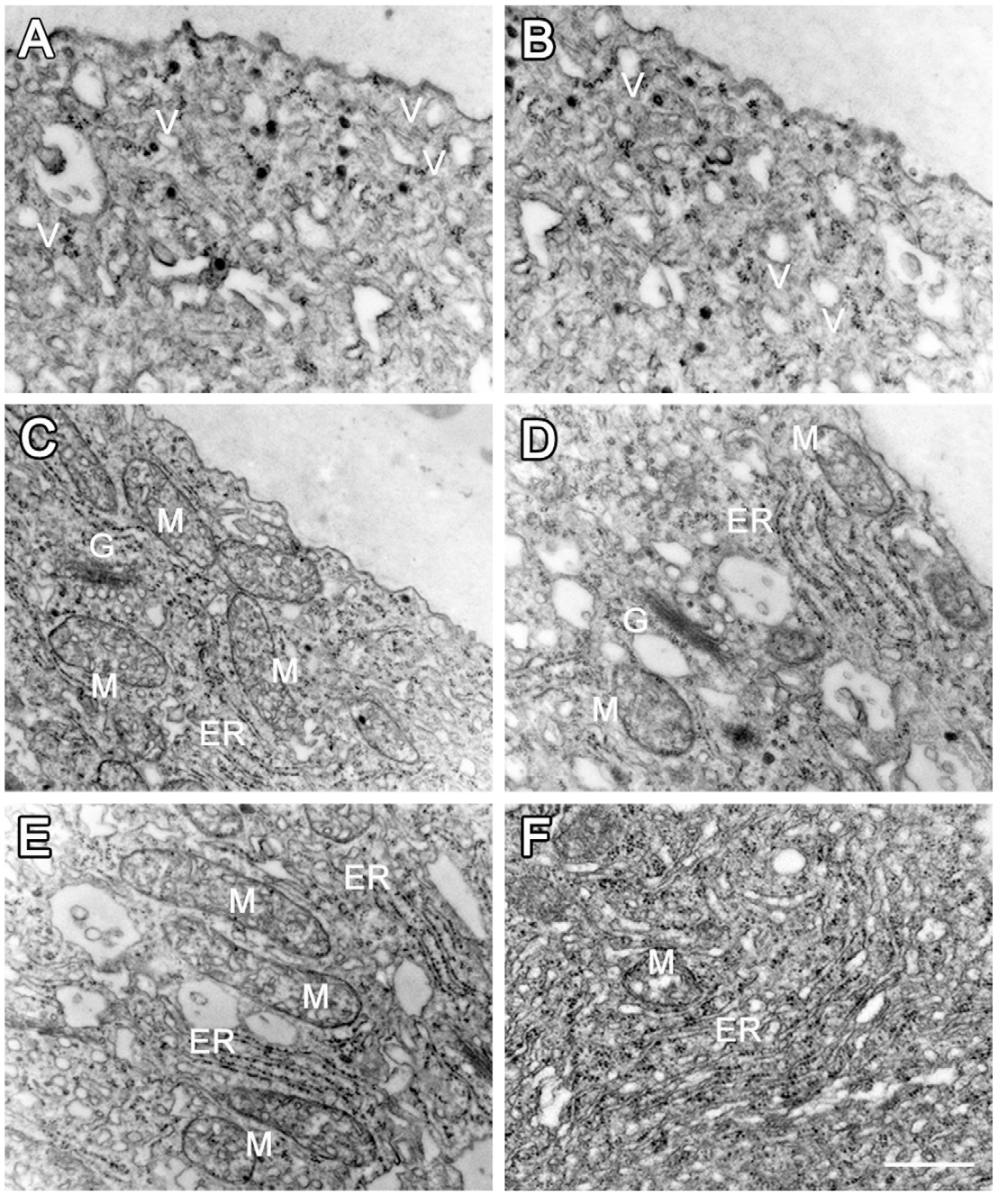
TEM analysis of cytoplasmic organization and organelle ultrastructure after anti-CaM treatment. A, Tip region of control pollen tube with abundant vesicles. B, Tip region in pollen tube after anti-CaM treatment, showing no obvious change in cytoplasmic organization. C, Mitochondria with electron-dense matrix and well-developed cristae in control pollen tube. D, Mitochondria without obvious abnormalities in ultrastructure upon anti-CaM treatment. E, Flat, tightly packed ERs with many attached ribosomes in control pollen tube. F, ERs with fewer attached ribosomes after anti-CaM treatment. Bar = 0.5 µm; G, Golgi stack; M, mitochondria; V, vesicle; ER, endoplasmic reticulum.

To test whether the tip region underwent changes after perturbation of the Ca^2+^ gradient, transmission electron microscopic (TEM) analyses were carried out to examine the ultrastructure of pollen tubes. In the control cells, organelles such as mitochondria, Golgi stacks, ER, and lipid droplets were accumulated in the subapical region, and the tip region was filled with vesicles (Figure 4A, 4C, 4E, *n* = 9). In contrast, the cytoplasmic organization in the tip region did not show obvious changes after applications of the pharmacological agents, except that fewer ribosomes were associated with rough ER (Figure 4B, *n* = 8). Larger organelles, such as mitochondria and Golgi stacks, were not significantly affected (Figure 4D, 4F).

### Protein Expression Profiles and Protein Identification

**Figure 5:**
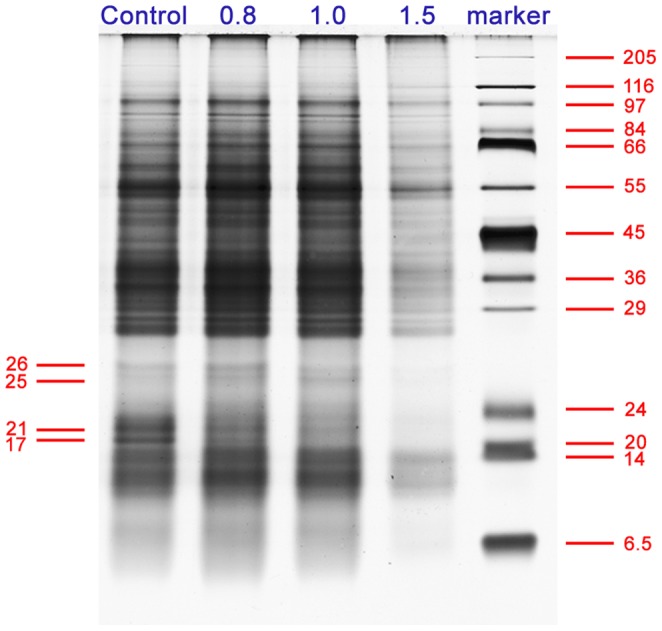
Protein expression profiles of *Cedrus deodara* pollen tubes cultured in the presence of different concentrations of anti-CaM (µg/mL). Proteins were extracted from pollen tubes incubated in standard medium (control) and in media containing 0.8, 1.0, or 1.5 µg/mL anti-CaM. Equivalent protein samples were separated by SDS-PAGE electrophoresis on 12% polyacrylamide gels (1 mm thick). Four bands showing significant changes in their expression levels were excised and subjected to further MS identification.

To detect proteins showing changes in abundance in response to dysfunction of apoplastic CaM, variations in the protein expression profile were analyzed. The amount of total proteins was similar among controls and the 0.8 and 1.0 µg/mL anti-CaM treatments, while there was a marked decrease in protein expression upon treatment with 1.5 µg/mL anti-CaM. In addition, there were pronounced decreases in several low molecular weight proteins after anti-CaM treatment (Figure 5). The expression levels of a 17-kDa protein and a 21-kDa protein continuously decreased with increasing anti-CaM concentration. In contrast, the expression levels of a 25-kDa protein and a 26-kDa protein increased by 47.79% and 51.81%, respectively, in the 0.8 µg/mL anti-CaM, showed a smaller increase in the 1.0 µg/mL anti-CaM treatment (22.73% and 11.76%, respectively), and decreased significantly in the presence of 1.5 µg/mL anti-CaM (as shown in Table S1).

To identify the proteins displaying differential expression, the protein bands of interest were excised from the sodium dodecyl sulfate polyacrylamide gel electrophoresis (SDS-PAGE) gel and subjected to in-gel digestion and tandem MS analysis. The 17-kDa band corresponded to a glycine-rich RNA-binding protein without a defined biological function (gi|4704605). The 21-kDa band matched to a previously unknown protein candidate. Further protein BLAST against NCBInr protein database showed that it had high homology to cyclophilin in *Picea sitchensis* (gi|116779193). Given that the genomic information for conifer species is still largely unresolved, the 25 kDa and 26 kDa bands also matched to putative protein candidates without definite annotations for their biological functions. Further protein BLAST showed the 25 kDa protein corresponded to a translationally controlled tumor protein (gi|116782446) and the 26 kDa protein corresponded to a phenylcoumaran benzylic ether reductase (gi|116784723) in *P. sitchensis* (Table 1).

**Table 1:** Identification of protein candidates showing significant changes in expression level in response to anti-CaM treatment.

ID.	Proteins identified	Accession No.	Organism	NCBI nr database		
				Mascot score	Number of matched peptides	Sequence coverage
17 KDa	Glycine-rich RNA-binding protein	gi|4704605	*Picea glauca*	401	6	48%
21 KDa	Unknown (putative cyclophilin)	gi|116779193	*Picea sitchensis*	136	4	15%
25 KDa	Unknown (putative translationally controlled tumor protein)	gi|116782446	*Picea sitchensis*	208	4	33%
26 KDa	Unknown (putative phenylcoumaran benzylic ether reductase)	gi|116784723	*Picea sitchensis*	656	10	51%

ID corresponds to protein bands indicated in Figure 5. Accession No. represents protein identified by MS and extracted from the National Center for Biotechnology Information (NCBI) database (in Aug, 2012). Mowse scores greater than 47 were considered significant (*p*<0.05); threshold score for individual ion was no less than 15.

### Variation in Distribution and Deposition of JIM5- and JIM7-reactive Pectins and Arabinogalactan Proteins during Apoplastic CaM Inhibition

**Figure 6:**
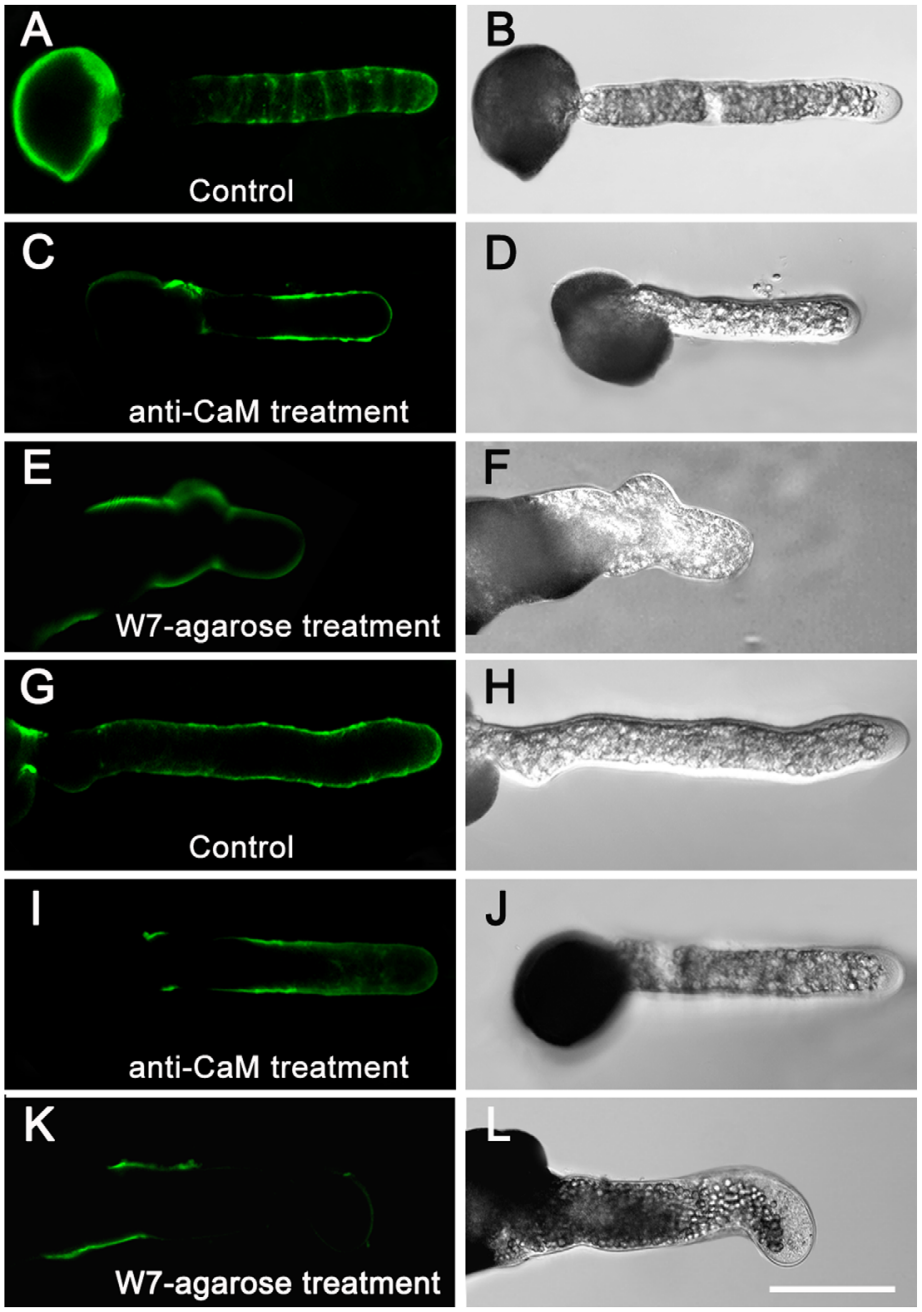
Variations in distribution and deposition of JIM5- and JIM7-reactive pectins after anti-CaM and W7-agarose treatments. Immunolabeling of pectins in pollen tube wall was carried out following the procedures described by Derksen (1999) with slight modifications. Pollen tubes were fixed and incubated in JIM5 and JIM7 as the primary antibody, and then incubated with FITC-labeled sheep anti-rat IgG as the secondary antibody. Samples were mounted and photographed under LSCM (excitation, 488 nm; emission, 522 nm). Controls were prepared by omitting the primary antibody. Samples consisted of 50 µL cultured pollen tubes (1 mg/mL at beginning of pollen culture) for immunofluorescence labeling experiments (repeated for three cultured sample bulks), of which five to nine pollen tubes were used for image collection and subsequent data analysis. A–B, JIM5-reactive pectins deposited in characteristic ring-like pattern along tube shank in control pollen tubes. C–D, JIM5-reactive pectins showed enhanced deposition along tube shank but slightly decreased deposition in apex, and characteristic ring-like deposition was no longer detected after anti-CaM application. E–F, W7-agarose application abolished characteristic ring-like deposition of JIM5-reactive pectins. G–H, JIM7-reactive pectins distributed almost uniformly along whole length of control pollen tubes. I–J, JIM7-reactive pectins displayed uneven distribution along the tube wall and fluorescence at the apex was relatively weak after anti-CaM treatment. K–L, Fluorescence of JIM7-reactive pectins was localized to confined region at basal site and only weak signal was detected at tip region after W7-agarose treatment. Bar = 50 µm.

In pollen tubes grown in the standard medium, JIM5 immunolabeling displayed a characteristic ring-like pattern of JIM5-reactive pectins in the tube shank along the walls of control pollen tubes (Figure 6A, 6B). After anti-CaM application, they showed enhanced deposition along the entire tube shank but slightly decreased deposition in the apex, and the characteristic ring-like deposition was no longer detected (Figure 6C, 6D). JIM7-reactive pectins fluorescence was distributed almost uniformly along the entire length of the control pollen tubes (Figure 6G, 6H), but was unevenly distributed along the tube wall after anti-CaM treatment and there was weak fluorescence at the apex (Figure 6I, 6J). W7-agarose induced similar variations in JIM5- and JIM7-reactive pectins (Figure 6E, 6F, 6K, 6L).

**Figure 7:**
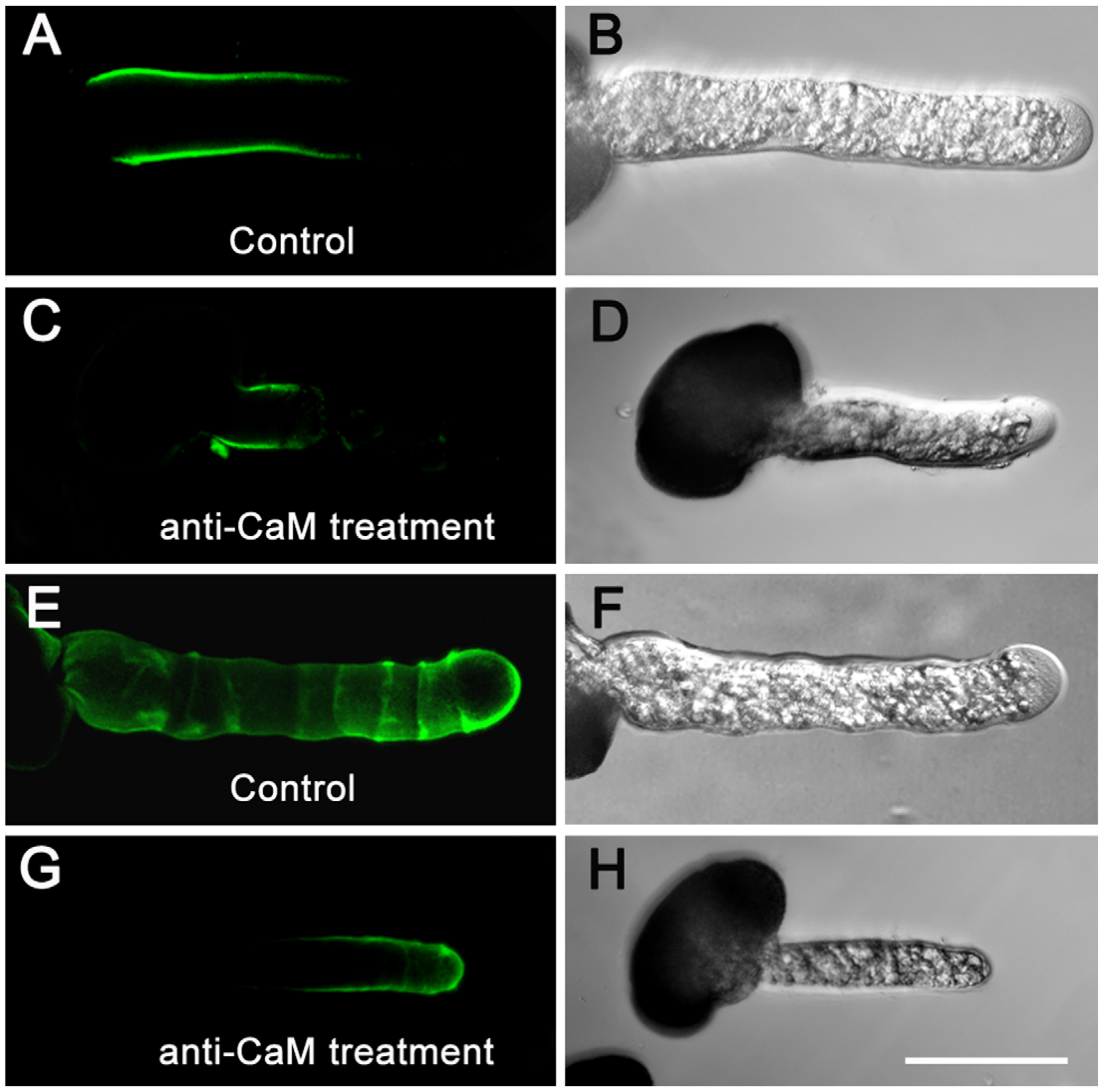
Deposition of LM2- and LM6-reactive AGPs in control and anti-CaM-treated pollen tubes. Immunolabeling of AGPs in pollen tube wall and subsequent image collection were carried out following procedures for pectin labeling except that LM2 and LM6 were used as primary antibodies. Pollen tubes were mounted and photographed under an LSCM (excitation, 488 nm; emission, 522 nm). Controls were prepared by omitting primary antibody. Samples consisted of 50 µL cultured pollen tubes (1 mg/mL at beginning of pollen culture) for immunofluorescence labeling experiments (repeated for three cultured sample bulks), of which five to nine pollen tubes were used for image collection and subsequent data analysis. A–B, Increasing gradient of LM2-reactive AGPs from subapical region to basal part of control pollen tubes. C–D, Fluorescence of LM2-reactive AGPs gradient was confined to basal part and did not extend further toward tip after anti-CaM treatment. E–F, LM6-reactive AGPs distributed in characteristic ring-like pattern similar to that of JIM5-reactive pectins along entire tube length of control pollen tubes. G–H, LM6-reactive AGPs were detected along tube shank and tip region after anti-CaM treatment, but characteristic ring-like pattern was abolished. Bar = 50 µm.

Immunolabeling of arabinogalactan proteins (AGPs) revealed that LM2-reactive AGPs formed a gradually increasing gradient from the subapical region to the basal part of the control pollen tubes (Figure 7A, 7B), while LM6-reactive AGPs were distributed in a characteristic ring-like pattern similar to that of JIM5-reactive pectins along the whole tube length in the control pollen tubes (Figure 7E, 7F). As a result, the gradient of LM2-reactive AGPs was not obvious after anti-CaM treatment but accumulated at the basal site and did not further extend to the tip (Figure 7C, 7D). The characteristic ring-like deposition of LM6-reactive AGPs was not detected after anti-CaM application (Figure 7G, 7H).

### Fourier Transform Infrared (FTIR) Spectroscopy

**Figure 8:**
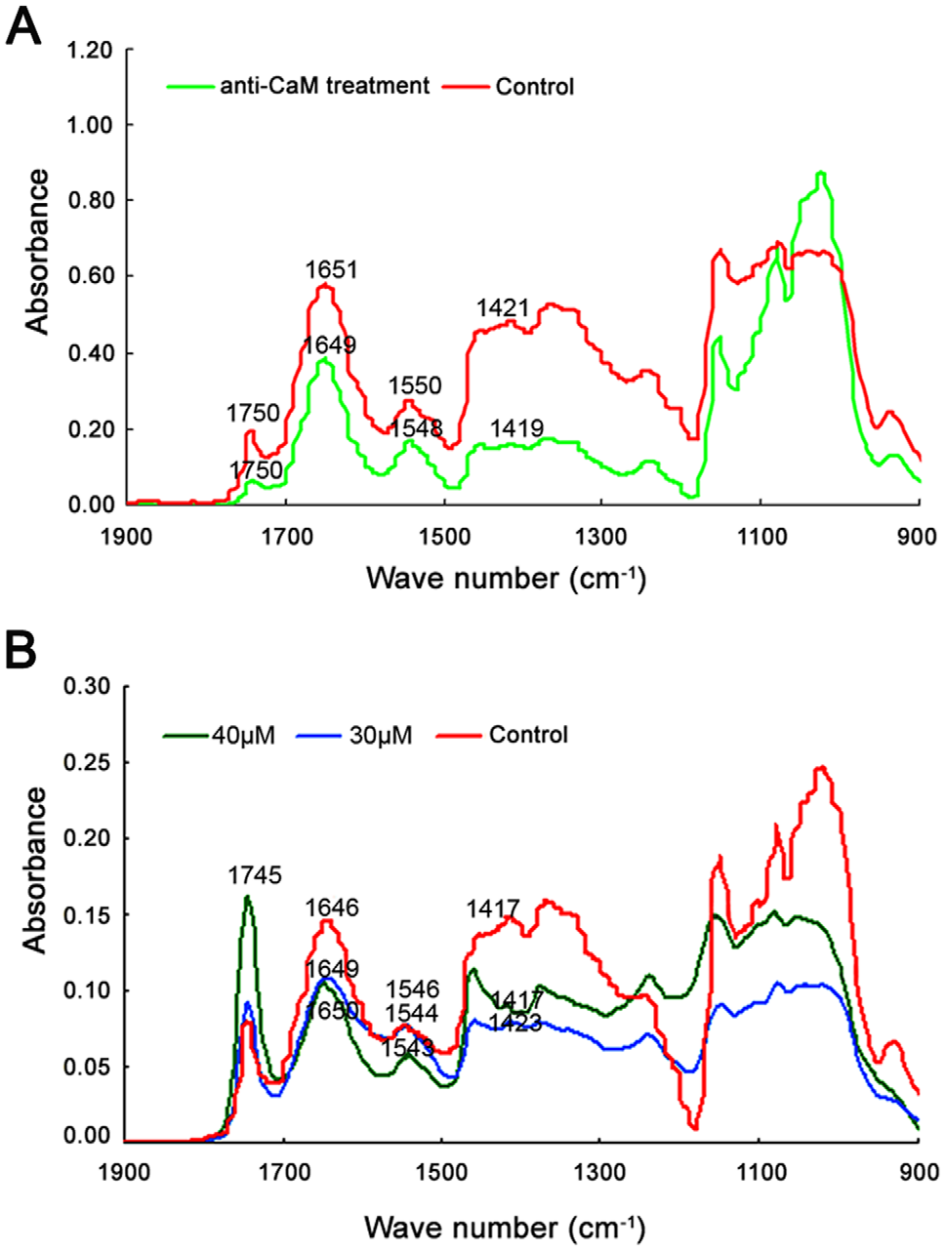
Fourier transform infrared spectra obtained from tip regions of *Cedrus deodara* pollen tubes. Three spectra were collected from each sample, and then averaged and baseline-corrected. Triplicate-averaged spectra were used to prepare figures shown in panels. A, FTIR spectra obtained from tip regions of pollen tubes incubated in the presence of 1.5 µg/mL anti-CaM. B, FTIR spectra obtained from tip regions of pollen tubes incubated in the presence of 30 and 40 µM W7-agarose. FTIR spectra showed that esterified pectins (saturated ester at 1745 cm^−1^) increased in tube wall while acidic pectins (carboxylic acid at 1415 cm^−1^) substantially decreased after treatments with pharmacological agents.

FTIR spectroscopy is a powerful and rapid assay for cell wall components and putative cross-links *in muro* [19], [20]. In the mid-IR spectrum, saturated esters absorb at 1740 cm^−1^ [21], amide-stretching bands of proteins absorb at 1650 and 1550 cm^−1^ [22], carboxylic acid groups absorb at 1600 and 1414 cm^−1^ [21], and carbohydrates absorb between 1200 and 900 cm^−1^ [23]. Highly reproducible FTIR spectra showed a marked change in the proportion of esterified and acid pectins after treatment with anti-CaM (Figure 8A). In the presence of 1.0 µg/mL anti-CaM, the ester peak at 1740 cm^−1^ increased, while free acid stretches at 1414 cm^−1^ decreased proportionally. The FTIR spectra were subjected to Gaussian fitting to quantify the relative contents of acidic and esterified pectins (Figure S7). The results showed that the ratio between acidic pectins and esterified pectins decreased from 3.86±0.77 (*n* = 5) to 2.59±0.43 (*n* = 7), indicating that acidic pectin deposition significantly decreased while esterified pectin increased upon anti-CaM treatment. W7-agarose treatments gave similar results and the changes in pectin deposition upon W7-agarose treatments were also analyzed using this method (Figure 8B). The results showed that the ratios after 30 and 40 µM W7-agarose treatments were 2.46±0.37 (*n* = 5) to 2.36±0.65 (*n* = 7), respectively, also implying decreased acidic pectin deposition and increased esterified pectin upon apoplastic CaM inhibition.

## Discussion

The pollen tube delivers male gametes to the egg in flowering plants and represents an ideal model system in which to investigate polarized growth in plant cells [1], [24]. To date, different complex signalling pathways have been reported to contribute to the tip-growth machinery, among which the Ca^2+^-CaM system is one of the most crucial [1], [5], [24], [25], [26].

It was reported that CaM was distributed evenly in the tip region of growing pollen tubes and the binding capacity of fluorescein isothiocyanate (FITC)-CaM was more intense in the sub-apical region, suggesting a higher concentration of CaM target molecules [25]. Rato *et al*. reported that CaM may be involved in polarized growth and re-orientation of pollen tubes by mediating ionic fluxes through cyclic-nucleotide-gated ion channels (CNGCs) and establishing the cross-talk machinery with multiple signalling pathways [26]. Similarly, previous studies also showed that apoplastic CaM can stimulate pollen germination and tube growth [9], [12], [15], [27]. During this process, one of the most important roles of CaM was to modulate the cytoplasmic Ca^2+^ concentration in plant cells [28]. Pharmacological analyses have been extensively used to investigate CaM functions [9], [11], [12], [28], [29]; for example, analyses of protein expression profiles showed differential primary responses and secondary responses upon application of the CaM antagonist trifluoperazine during pollen germination and tube growth [29]. In the present study, two pharmacological agents, anti-CaM and W7-agarose, were effectively prevented from passing through the plasma membrane. Therefore, the results demonstrated here that anti-CaM and W7-agarose substantially disturbed the normal functions of apoplastic CaM and subsequently caused morphological abnormalities and/or blocked growth in *C. deodara* pollen tubes. During this process, an elaborate mechanism may be employed, whereby apoplastic CaM responded to extracellular stimuli and transduced the signal through the plasma membrane, subsequently causing intracellular events leading to changes in the concentrations and distributions of macromolecules.

It has been reported that calcium, a universal secondary messenger, mediates coupling of the stimulus-triggered responses to regulations of diverse cellular functions during pollen tube development, among which the calcium influx and the tip-focused calcium gradient are indispensable [30]–[34]. A tip-to-base cytoplasmic [Ca^2+^]_c_ gradient was demonstrated in growing pollen tubes of *Lilium longiflorum* and *P. abies* [30], [35], [36]. This gradient was largely maintained by extracellular Ca^2+^ influx and was considered to be relevant in regulating the growth and orientation of growing cells [35], [36]. In the present study, CaM antagonists rapidly disrupted the tip-focused cytosolic calcium gradient along the growing pollen tubes within only a couple of minutes, particularly affecting the steepness of gradient. This indicated that dissipation of the Ca^2+^ gradient was a rapid primary change in response to the dysfunction of apoplastic CaM, and that the perturbation in Ca^2+^ homeostasis was one of the early events. Considering that the tip-focused cytosolic calcium in pollen tubes was mainly maintained by extracellular Ca^2+^ influx [37], [38], we proposed that apoplastic CaM was involved in the intricate regulation of pollen tube development by maintaining the Ca^2+^ gradient and modulating Ca^2+^ homeostasis. In addition, we noticed certain aberrant morphological changes, for example, branched tubes and expanded tubes with swollen tips, after treatment with W7-agarose. These changes may be attributed to the high specificity for the naphthalene ring of W7-agarose as the largest that can fit into the hydrophobic binding pockets of Ca^2+^-CaM [39]. Given that apoplastic CaM can dramatically affect the pattern of cytoplasmic free calcium in pollen tube tips, it seems reasonable to conclude that the decreases in germination rate and tube elongation rate may result from abnormal growth due to perturbation of the tip-focused cytoplasmic [Ca^2+^]_c_ gradient.

CaM, the predominant calcium receptor, is one of the best-characterized calcium sensors regulated by Ca^2+^ binding in eukaryotes [12], [25], [29], [40], [41]. It has been assumed that certain CaM-interacting proteins act as signal receptors on the plasma membrane and that the Ca^2+^-CaM complex regulates its targets by manipulating CaM effectors/transducers [13], [15]. Our TEM observations showed that application of pharmacological agents did not significantly affect the cytoplasmic organization, except that there were fewer ribosomes associated with rough ER, implying potential changes in protein synthesis activity. To further detect proteins involved in the responses to dysfunction of apoplastic CaM, SDS-PAGE electrophoresis and identification of differentially expressed protein bands were carried out. The 21-kDa protein corresponded to a putative member of the cyclophilin family (gi|116779193), whose expression level continuously decreased with increasing anti-CaM concentration (Table S1). Indeed, it was previously reported by Yokota *et al*. that significant amounts of a 21-kDa cyclophilin were specifically released into the extracellular medium when pollen grains of *L. longiflorum* were incubated with EGTA or at low Ca^2+^ concentrations [42]. Alternatively, the decrease in the expression level of this protein may have resulted from secondary effects of reduced germination and growth activity. We proposed that the decrease in the 21-kDa cyclophilin might be involved in the responses to the blockage of apoplastic CaM signalling. Moreover, a band at about 17 kDa was absent from all of the anti-CaM treatments, whereas two bands at about 25 and 26 kDa were barely detectable in the 1.5 µg/mL anti-CaM treatment. The 17, 25, and 26 kDa proteins were identified as a glycine-rich RNA-binding protein (gi|4704605), a putative translationally controlled tumor protein (gi|116782446) and a phenylcoumaran benzylic ether reductase (gi|116784723), respectively. The biological functions of these proteins in the context of polarized growth of pollen tubes have not been elucidated. These results further suggested that proteins of multiple functional categories may respond differentially to perturbations in apoplastic CaM signalling.

It has been demonstrated that the characteristic deposition and dynamic transformation of pectins and AGPs are crucial elements for polarized cell growth [43]–[46]. The observed ring-like deposition of pectins was reported to be correlated with oscillatory or pulsatory growth in pollen tubes, a phenomenon also documented in angiosperm pollen tubes [43], [45]. Moreover, the functions of pectin methyl esterase (PME) with respect to methylesterified pectins secreted at the growing apex have been analyzed [47]. The degree of esterification is thus essential for cell wall mechanics as unesterified pectins are able to bind calcium ions, resulting in the gelation of polymers. This process can rigidify the wall as revealed by micro-indentation [45]. Given that the mechanical characteristics of the cell wall are important for sustained polarized growth [47], [48], [49], the results from immunolabeling of cell wall components demonstrated that different configurations of pectins and AGPs showed altered depositions and distribution in the cell wall, especially in the tip region, after application of inhibitors. This finding indicated that blockage of apoplastic CaM triggered a dramatic remodeling of cell wall components at the tip, which contributed to substantial mechanistic changes in cell wall rigidity/extensibility, and subsequent slowing and cessation of growth.

FTIR spectroscopy is a reliable method to investigate cell wall components [19], [20], and it has been used to analyze various chemical components during pollen tube growth in different media [29], [44]. The present study revealed that anti-CaM- and W7-agarose-treated pollen tubes showed increased esterified pectin (saturated ester absorbing at 1740 cm^−1^) and decreased acidic pectin (carboxylic acid absorbing at 1415 cm^−1^) compared with those in normal pollen tubes. The decrease in the ratio between acidic pectins and esterified pectins further implied decreased rigidity and increased extensibility upon apoplastic CaM inhibition. These results were consistent with our immunolabeling results and earlier findings in other cell types [50], [51]. The rigidity and/or extensibility of cell walls in carrot cells were altered by changes in apoplastic Ca^2+^ concentrations [50], giving rise to substantial changes in the mechanistic properties of cell walls by binding pectins into a network of “egg-crate” linkages [51]. Given the link between extracellular CaM and the variation in pectin contents, this is an important finding suggesting that apoplastic CaM might be involved in regulating the transformation between different pectin configurations. The inhibition of apoplastic CaM may perturb its interaction with Ca^2+^, further affecting the production of acidic pectins. This would result in loosening the cell wall, which could be visualized as disrupted and swollen pollen tubes.

In conclusion, apoplastic CaM contributed to polarized growth of pollen tubes by modulating cytoplasmic calcium homeostasis and cell wall modeling. Anti-CaM and W7-agarose antagonized apoplastic CaM, which led to rapid perturbation in the steepness of the tip-to-base Ca^2+^ gradient, giving rise to changes in the concentration and distribution of carboxylic acid and saturated esters, as well as changes in expression profiles of several proteins. The interaction between Ca^2+^ and apoplastic CaM might be central to the maintenance of calcium gradients, cell wall modeling, and other aspects of pollen tube growth.

## Materials and Methods

### Plant Materials

Pollen grains were collected from *C. deodara* (Roxb.) Loud. trees growing in the Botanical Garden of the Institute of Botany, Chinese Academy of Sciences, on May 10, 2010, when most of the cones were ripe. The collected pollen cones were blotted dry on paper towels and then kept in Petri dishes for 3–5 d at room temperature. Dried pollen grains were stored in vials at −20°C until use.

### Pollen Culture Conditions

The standard medium for pollen germination and tube growth contained 15% (w/v) sucrose, 0.01% H_3_BO_3_, and 0.01% CaCl_2_ adjusted to pH 6.8. Stored pollen grains were equilibrated at room temperature for 30 min and dispersed in the culture medium to a final concentration of 1.5 mg/mL. The CaM monoclonal antibody and W7-agarose (Sigma, St. Louis, MO, USA) were added to the culture medium at various concentrations at the beginning of incubation. Mouse serum (Sigma) controls for anti-CaM were treated in the same manner. The suspension was shaken on a rotary shaker (100 rpm) at 25°C in the dark.

### Observations of Pollen Germination and Tube Growth

Pollen grains were suspended in culture media supplemented with 0, 0.8, 1, or 2 µg/mL anti-CaM or 0, 20, 40, or 60 µM W7-agarose. Pollen germination rates were determined and the lengths of pollen tubes were measured microscopically after 5–7 d of incubation, depending on the experiment. Pollen grains were considered to be germinated when the tubes grew longer than the diameter of the pollen grain (Dafni, 2000). For germination rates, 500–600 pollen grains were counted; for pollen tube growth, 50–60 tubes were measured per replicate, with three replicates per treatment.

### Microinjection of Calcium Green-1 Dextran

Pollen tubes were kept on a coverslip attached to the bottom of a microscope slide chamber with a thin layer of media supplemented with 1% agarose (type VII; Sigma). Microinjection was performed under an Axiovert 200 M inverted microscope (Eppendorf TransferMan NK2, Germany). For microinjections, 2.5 mM Calcium Green-1 dextran (CG-1D, 10 000 MW, Molecular Probes Inc., Eugene, OR) in 5 mM HEPES buffer, pH 7.0, was pressure-injected into the pollen tube as described previously [52]. The Ca^2+^ dynamics of the injected pollen tubes was subsequently recorded using a LSM 510 META LSCM (Zeiss Co., Germany) in time-course mode after corresponding treatments with agents.

### Electron Microscopy

Pollen tubes were collected and fixed for 2 h in 2.5% glutaraldehyde in 100 mM phosphate buffer (pH 7.2) with 2% (w/v) sucrose, then washed with 100 mM phosphate buffer, post-fixed in 2% osmium tetroxide for 2 h, dehydrated in an ethanol series, and embedded in Spurr’s resin. Sections were cut using an LKB-V ultramicrotome, stained with 2% uranyl acetate (w/v) in 70% methanol (v/v) and 0.5% lead citrate, and then examined under a JEM-1230 electron microscope (JEOL Ltd., Japan).

### SDS-PAGE and Protein Identification

Frozen pollen tubes were ground to fine powder and resuspended in 65 mm Tris-HCl buffer (pH 6.8), 1% SDS, 5% glycerol, and 2.5% mercaptoethanol as described previously [53]. The suspension was boiled for 5 min and centrifuged at 14000 *g* for 30 min. The protein concentrations were measured according to Schaffner & Weismann (1973) with bovine serum albumin as a standard [54]. Equivalent protein samples were subjected to SDS-PAGE electrophoresis on 12% polyacrylamide gels (1 mm thick) according to Laemmli (1970) [55]. Low molecular mass markers (14.4–97 kDa, Amersham BioSciences, Uppsala, Sweden) were used as standards, and the gel was silver-stained. Semi-quantitative analysis was carried out for bands of interest using gray-scale measurements with Image J software.

The bands of interest were excised from the gel and subjected to in-gel digestion and CapLC Q-TOF MS/MS analysis. Full descriptions of these procedures are given in the Supplemental Data. The digested protein solutions were desalted with ZipTip C18 pipette tips (Millipore), and analyzed by ESI-Q time-of-flight tandem mass spectrometry (MS/MS; Micromass). Database searching was performed using the Mascot search engine (www.matrixscience.com). To qualify as positive identification, the following criteria were used: database, NCBI nr; taxonomy, Viridiplantae (green plants); one missed cleavage was allowed; peptide tolerance, 1.2; MS/MS tolerance, 0.5; enzyme, trypsin; modifications (such as carbamidomethyl and oxidation) were used.

### Confocal Observation of Distribution of JIM5-, JIM7-, LM2-, and LM6-reactive Epitopes in Pollen Tube Wall

Immunolabeling of pectins and AGPs in the pollen tube wall was carried out following the procedures described by Chen *et al*. (2009) [29]. Monoclonal antibodies JIM5 and JIM7 were used to label JIM5- and JIM7-reactive pectins, respectively, and monoclonal antibodies LM2 and LM6 were used to localize AGPs epitopes. At 72 h after germination, pollen tubes were fixed in 3% formaldehyde in PME buffer (50 mmol/L PIPES, 0.5 mmol/L MgCl_2_, 1 mmol/L EGTA, pH 6.8) for 30 min at room temperature. After three washes with PME buffer and one wash with phosphate-buffered saline (PBS; pH 7.2), the samples were incubated for 2.5 h at room temperature with primary antibodies. After incubation, pollen tubes were washed with PBS three times and then incubated with FITC-labeled sheep anti-rat IgG (Sigma) diluted 1∶100 with PBS for at least 2 h at room temperature. Then, they were washed with PBS three times, mounted, and photographed under a Zeiss laser scanning confocal microscope as described above, except that excitation was at 488 nm and emission at 522 nm. Controls were prepared by omitting the primary antibody. For the immunofluorescence labeling experiments, 50 µL cultured pollen tubes (1 mg/mL at the beginning of pollen culture) was used (from three separate pollen cultures). From each sample, seven to nine pollen tubes were used for subsequent data analysis.

### FTIR Spectroscopy Analysis of Wall Components

Pollen tubes were cultured in standard medium supplemented with 1.5 µg/mL anti-CaM and 40 µM W7-agarose (Sigma) for 3 d, then centrifuged at 500 rpm for 2 min and washed three times with deionized water. The pollen tubes were mounted on a barium fluoride window on the stage of the Nicolet NicPlan IR Microscope accessory of the Nicolet Magna-IR 750 FTIR spectrometer equipped with a liquid nitrogen-cooled mercury cadmium telluride detector. An area of the cell wall (100×100 µm) in the tip region was selected for spectral collection. Sixty-four interferograms were collected in transmission mode with a 4 cm^−1^ resolution and were co-added together to improve the signal-to-noise ratio. Three spectra were collected from each sample, and then averaged and baseline-corrected. The triplicate-averaged spectra were then used to prepare figures shown in panels.

## Supporting Information

Figure S1 **Monoclonal anti-green fluorescent protein (GFP) antibody did not have obvious inhibitory effects on pollen germination and tube elongation, while exogenous CaM partly recovered the inhibition.** Monoclonal anti-green fluorescent protein (GFP) antibody was used as a control to test its effects on pollen germination and tube elongation. Exogenous calmodulin was also applied in two different concentrations (10^−9^ and 10^−8^ mol/L) to compete against anti-CaM.
(TIF)

Figure S2 **Rinsing solution of W7-agarose did not have obvious inhibitory effects on pollen germination and tube elongation, while beads after rinsing retained inhibitory effects.** W7 agarose beads were extensively rinsed with culture solution using the same experimental procedures used for pollen germination (beads were extensively rinsed with culture solution by shaking on a rotary shaker at 100 rpm and 25°C in the dark for 30 min), and then effects of the washed beads and their washing fluid on pollen germination and tube elongation were evaluated. Exogenous calmodulin (10^−9^ and 10^−8^ mol/L) was also included in the experiment to outcompete the effects of W7 agarose.
(TIF)

Figure S3 **Dysfunction of apoplastic CaM rapidly disturbed cytoplasmic [Ca^2+^]_c_ gradient.** A, Example of measuring variations in cytoplasmic [Ca^2+^]_c_ gradient induced by anti-CaM. For each tube, reduction in calcium gradient was measured by calculating ratio of fluorescence of cytoplasmic free calcium at extreme apex to that at base of clear zone (red dashed line indicates direction in which fluorescence intensity was measured). B, Pollen tubes showed an obvious tip-to-base cytoplasmic [Ca^2+^]_c_ gradient (fluorescence intensity is represented by gray scale value). C, Pollen tubes cultured in standard medium showed almost 3-fold tip-focused gradient (2.57±0.19, *n* = 9), while pollen tubes cultured in medium containing 1.0 µg/mL anti-CaM showed significantly shallower calcium gradient (1.55±0.05, *n* = 7).
(TIF)

Figure S4 **Confocal images of cytosolic free calcium distribution in control and anti-CaM treatment pollen tube (displayed in rainbow mode).** Pollen tubes cultured in treatment and control were loaded with 20 µM Fluo-3/AM ester at 4°C for 2 h in the dark, then rinsed and kept at room temperature in dark for 1 h. Samples of treatments with anti-CaM and controls were collected, mounted, and photographed under a Zeiss LSM 510 Meta LSCM (Zeiss Co., Germany) (excitation, 488 nm; emission, 515 nm). A, Pollen tube cultured in standard medium showing steep gradient of cytosolic free calcium in growing tip. B, Pollen tube incubated with 0.8 µg/mL anti-CaM showing altered calcium distribution with much shallower gradient. C, Pollen tube treated with 1.0 µg/mL anti-CaM showing completely disrupted calcium distribution in cytoplasm instead of tip-focused Ca^2+^ gradient. Original fluorescence images and bright field images are shown in small box. D–G, Dissipation of tip-focused gradient within a few seconds under anti-CaM treatment. Bar = 50 µm.
(TIF)

Figure S5 **Time course analysis of [Ca^2+^]_c_ changes upon 30 µM W7-agarose treatment.** Calcium Green-1 Dextran microinjection was carried out to further elucidate the changes in calcium gradient upon addition of W7-agarose. Experiments were performed according to the procedures in Figure 3 except that W7-agarose was applied at 60 s. Control pollen tubes showed a typical tip-focused [Ca^2+^]_c_ gradient (A–B), whereas [Ca^2+^]_c_ gradient rapidly became shallower by approximately 160 s after W7-agarose application (E–L). Bar = 20 µm.
(TIF)

Figure S6 **Measurement of net calcium flux at extreme tip region in response to inhibition on CaM by non-invasive scanning ion-selective electrode technique (SIET).** Net Ca^2+^ flux was measured at Xu-Yue Sci. & Tech. Co., Ltd., using SIET. Excel software was used to convert data obtained by ion selective probe technique from background -mV estimation of concentration and microvolt difference estimation of local gradient into specific ion influx (pmol cm^−2^ s^−1^). A, Left, Noninvasive scanning ion-selective electrode test showed that 1.5 µg/mL anti-CaM induced a rapid increase in extracellular Ca^2+^ influx. Right, mean values for net Ca^2+^ influxes before and after treatments with anti-CaM (*n* = 10). B, Left, Noninvasive scanning ion-selective electrode test showed that 30 µM W7-agarose induced a rapid increase in extracellular Ca^2+^ influx and then it fluctuated between influx and efflux. Right, mean values for net Ca^2+^ influxes before and after treatments withW7-agarose (*n* = 7).
(TIF)

Figure S7 **Gaussian fitting for FTIR spectra to quantify relative contents of esterified and acidic pectins.** A, Gaussian fitting results for FTIR spectra for control cells (r^2^ = 0.98613). B, Gaussian fitting results for FTIR spectra for pollen tubes treated with 1.5 µg/mL anti-CaM (r^2^ = 0.98957). FTIR spectra were subjected to Gaussian fitting to quantify relative contents of acidic and esterified pectins using Origin 9.0 software (Origin Lab). Ratio between esterified pectins and acidic pectins was calculated from percentages of characteristic peak for esterified pectins and characteristic peak for acidic pectins. Ratio between acidic pectins (light blue peak) and esterified pectins (yellow peak) decreased from 3.86±0.77 (n = 5) to 2.59±0.43 (n = 7), indicating that acidic pectin deposition significantly decreased while esterified pectin increased upon anti-CaM treatment. Proportion of protein peaks (peaks at 1650 and 1550 cm^−1^) to total absorption peaks was also calculated, giving values of 0.49±0.12 (*n* = 5) for control cells and 0.35±0.19 (*n* = 7) for anti-CaM treated pollen tubes.
(TIF)

Table S1 **Semi-quantitative analysis for the interested protein bands in SDS-PAGE gels.** The results were obtained from three independent SDS-PAGE gels by semi-quantitative analysis for the gray scale values from the interested protein bands with Image J software.
(DOC)

File S1 **Supplemental materials and methods for recovery test in** **Figure 1** **, calcium influx measurement and Fluo-3/AM staining.**
(DOC)
