# Supplementary figures and images for: Inhibition of Apoplastic Calmodulin Impairs Calcium Homeostasis and Cell Wall Modeling during Cedrus deodara Pollen Tube Growth
Source: PLoS One. 2013 Feb 6;8(2):e55411. doi: 10.1371/journal.pone.0055411 (PMC3566176; doi:10.1371/journal.pone.0055411)

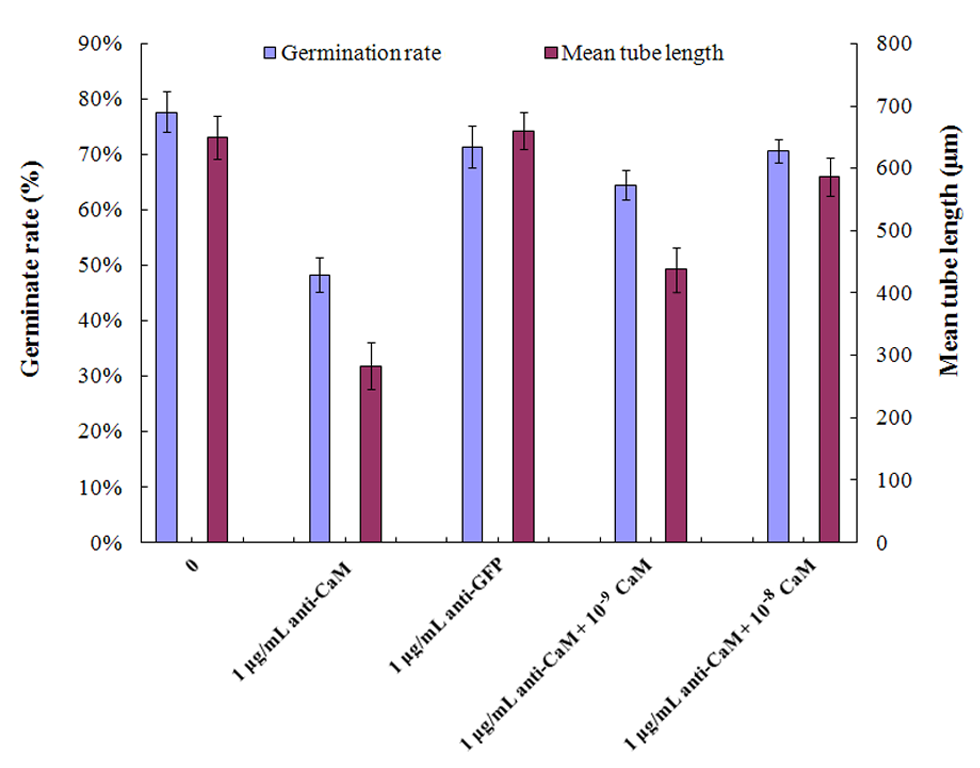

Supplement: Figure S1 — Monoclonal anti-green fluorescent protein (GFP) antibody did not have obvious inhibitory effects on pollen germination and tube elongation, while exogenous CaM partly recovered the inhibition. Monoclonal anti-green fluorescent protein (GFP) antibody was used as a control to test its effects on pollen germination and tube elongation. Exogenous calmodulin was also applied in two different concentrations (10−9 and 10−8 mol/L) to compete against anti-CaM. (TIF) [file pone.0055411.s001.tif]

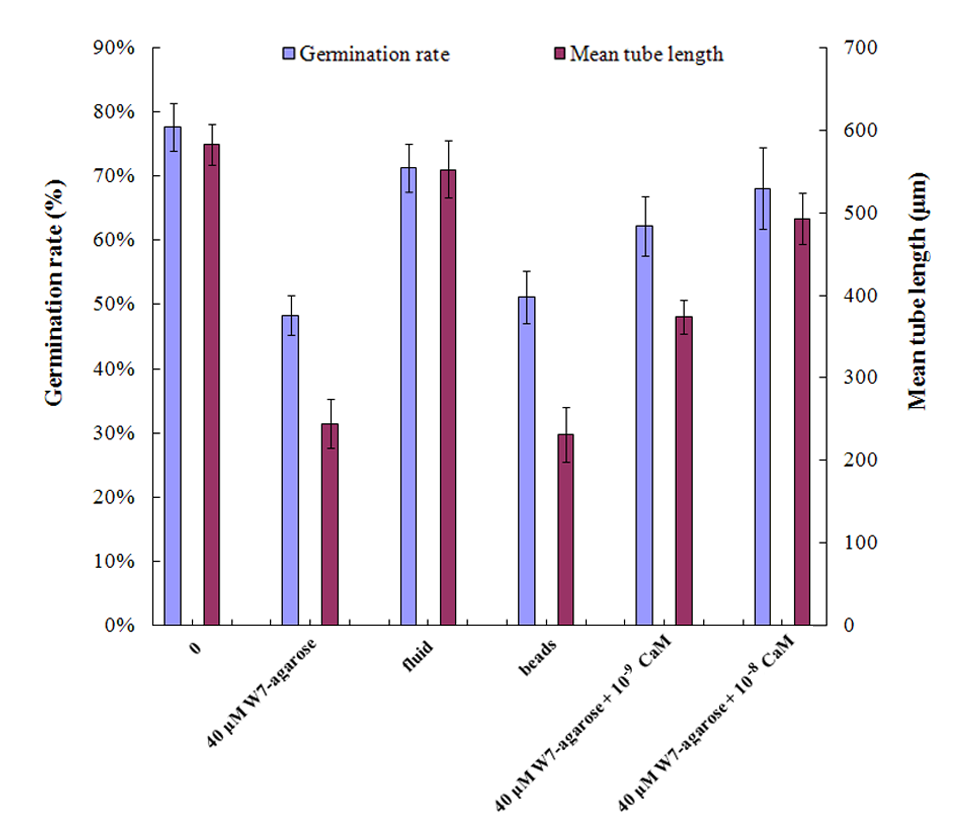

Supplement: Figure S2 — Rinsing solution of W7-agarose did not have obvious inhibitory effects on pollen germination and tube elongation, while beads after rinsing retained inhibitory effects. W7 agarose beads were extensively rinsed with culture solution using the same experimental procedures used for pollen germination (beads were extensively rinsed with culture solution by shaking on a rotary shaker at 100 rpm and 25°C in the dark for 30 min), and then effects of the washed beads and their washing fluid on pollen germination and tube elongation were evaluated. Exogenous calmodulin (10−9 and 10−8 mol/L) was also included in the experiment to outcompete the effects of W7 agarose. (TIF) [file pone.0055411.s002.tif]

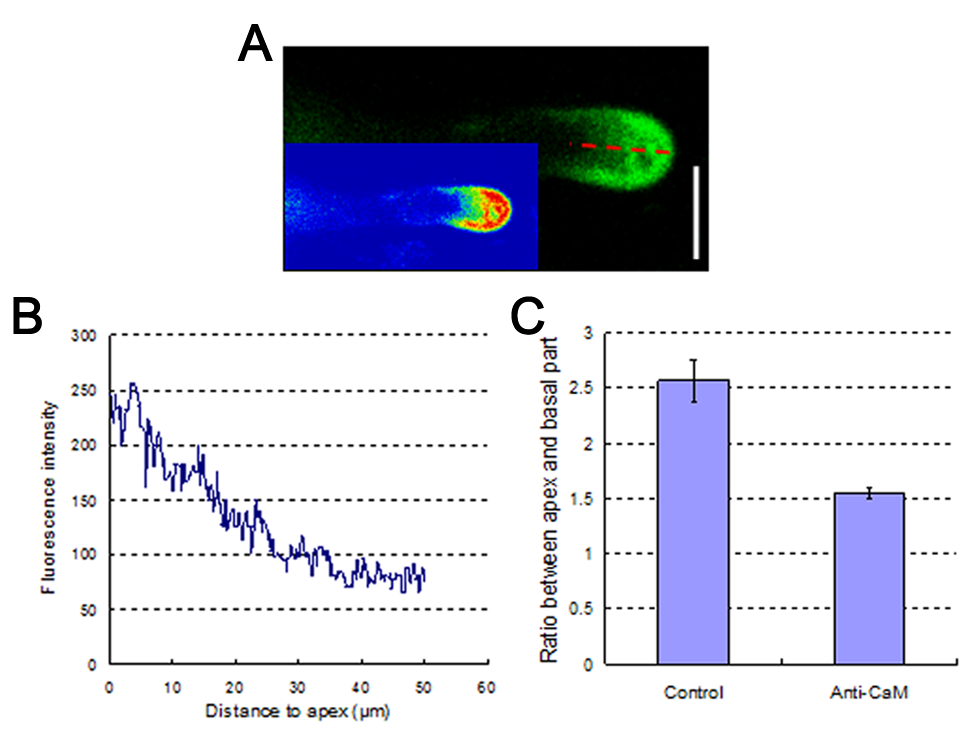

Supplement: Figure S3 — Dysfunction of apoplastic CaM rapidly disturbed cytoplasmic [Ca2+]c gradient. A, Example of measuring variations in cytoplasmic [Ca2+]c gradient induced by anti-CaM. For each tube, reduction in calcium gradient was measured by calculating ratio of fluorescence of cytoplasmic free calcium at extreme apex to that at base of clear zone (red dashed line indicates direction in which fluorescence intensity was measured). B, Pollen tubes showed an obvious tip-to-base cytoplasmic [Ca2+]c gradient (fluorescence intensity is represented by gray scale value). C, Pollen tubes cultured in standard medium showed almost 3-fold tip-focused gradient (2.57±0.19, n = 9), while pollen tubes cultured in medium containing 1.0 µg/mL anti-CaM showed significantly shallower calcium gradient (1.55±0.05, n = 7). (TIF) [file pone.0055411.s003.tif]

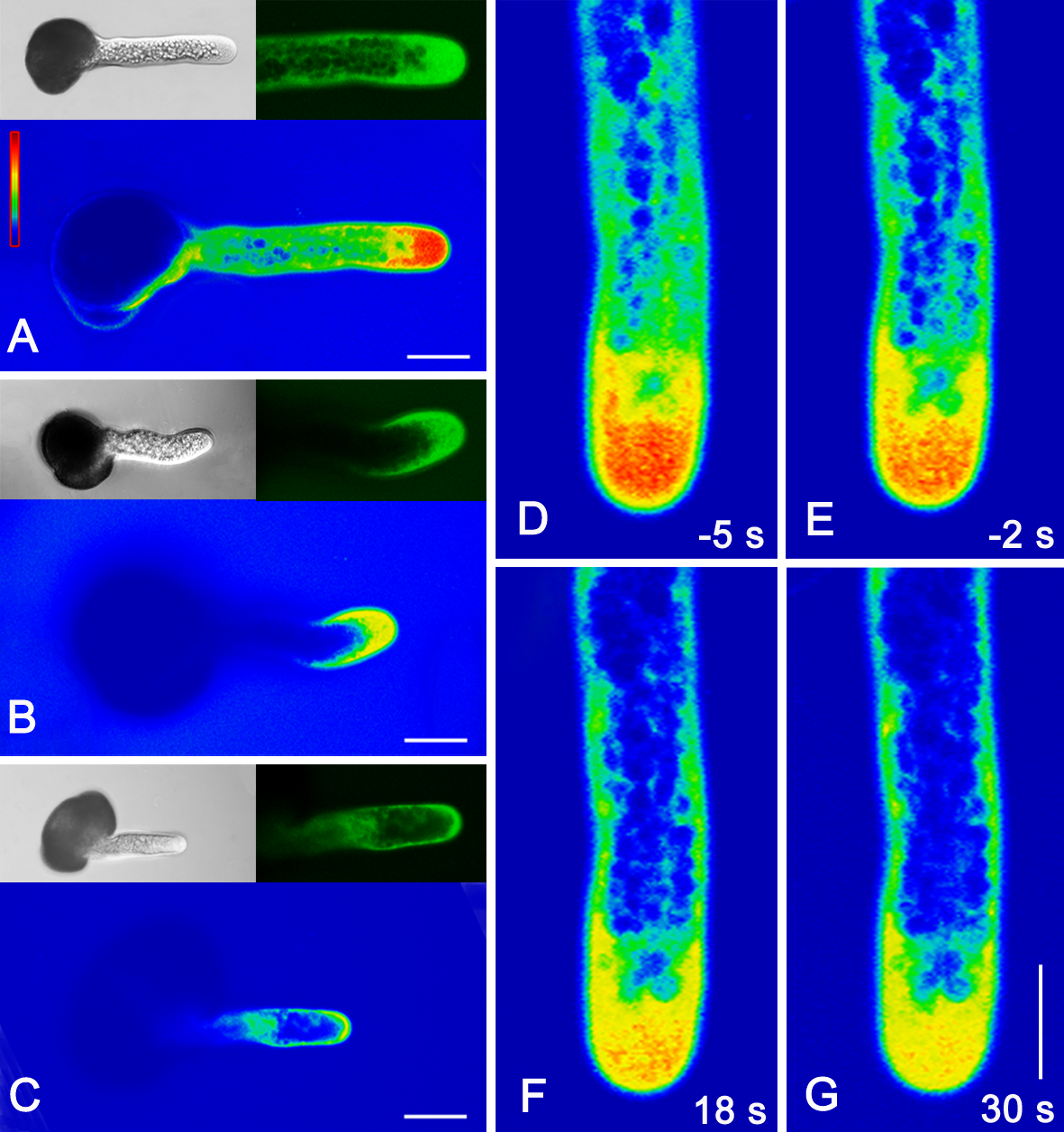

Supplement: Figure S4 — Confocal images of cytosolic free calcium distribution in control and anti-CaM treatment pollen tube (displayed in rainbow mode). Pollen tubes cultured in treatment and control were loaded with 20 µM Fluo-3/AM ester at 4°C for 2 h in the dark, then rinsed and kept at room temperature in dark for 1 h. Samples of treatments with anti-CaM and controls were collected, mounted, and photographed under a Zeiss LSM 510 Meta LSCM (Zeiss Co., Germany) (excitation, 488 nm; emission, 515 nm). A, Pollen tube cultured in standard medium showing steep gradient of cytosolic free calcium in growing tip. B, Pollen tube incubated with 0.8 µg/mL anti-CaM showing altered calcium distribution with much shallower gradient. C, Pollen tube treated with 1.0 µg/mL anti-CaM showing completely disrupted calcium distribution in cytoplasm instead of tip-focused Ca2+ gradient. Original fluorescence images and bright field images are shown in small box. D–G, Dissipation of tip-focused gradient within a few seconds under anti-CaM treatment. Bar = 50 µm. (TIF) [file pone.0055411.s004.tif]

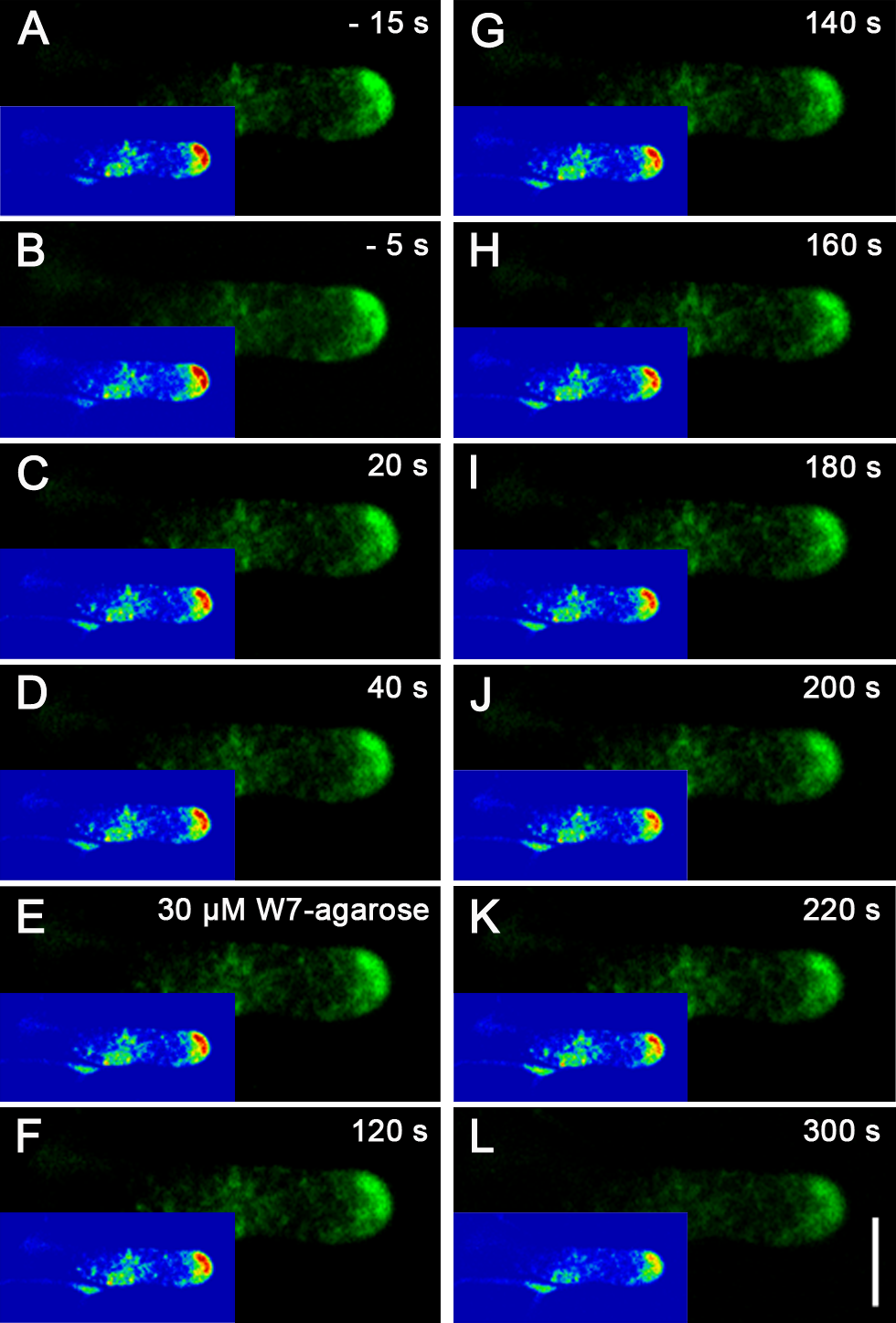

Supplement: Figure S5 — Time course analysis of [Ca2+]c changes upon 30 µM W7-agarose treatment. Calcium Green-1 Dextran microinjection was carried out to further elucidate the changes in calcium gradient upon addition of W7-agarose. Experiments were performed according to the procedures in Figure 3 except that W7-agarose was applied at 60 s. Control pollen tubes showed a typical tip-focused [Ca2+]c gradient (A–B), whereas [Ca2+]c gradient rapidly became shallower by approximately 160 s after W7-agarose application (E–L). Bar = 20 µm. (TIF) [file pone.0055411.s005.tif]

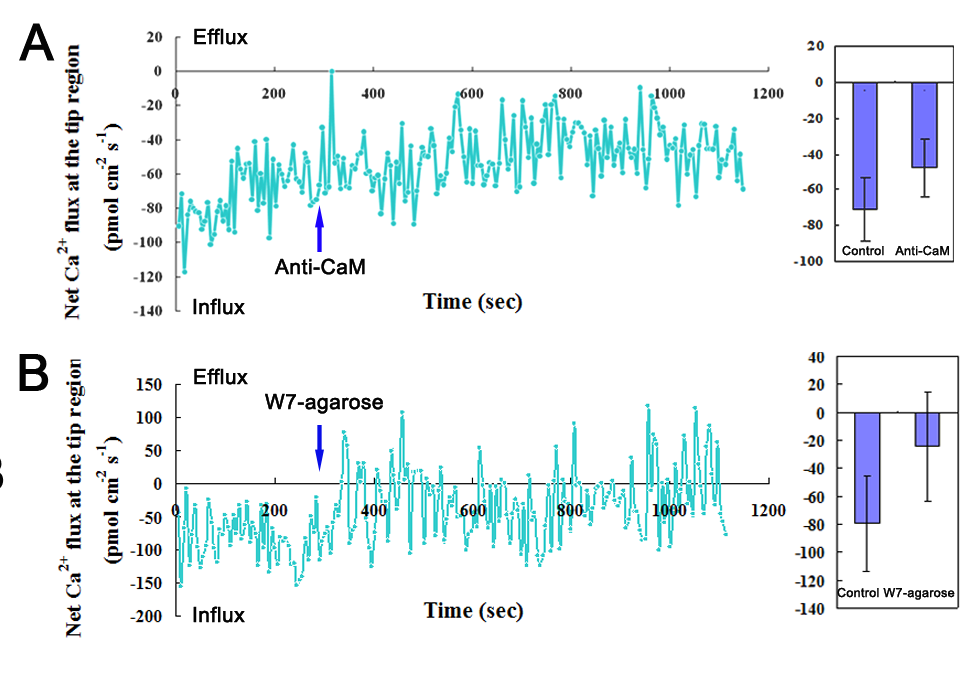

Supplement: Figure S6 — Measurement of net calcium flux at extreme tip region in response to inhibition on CaM by non-invasive scanning ion-selective electrode technique (SIET). Net Ca2+ flux was measured at Xu-Yue Sci. & Tech. Co., Ltd., using SIET. Excel software was used to convert data obtained by ion selective probe technique from background -mV estimation of concentration and microvolt difference estimation of local gradient into specific ion influx (pmol cm−2 s−1). A, Left, Noninvasive scanning ion-selective electrode test showed that 1.5 µg/mL anti-CaM induced a rapid increase in extracellular Ca2+ influx. Right, mean values for net Ca2+ influxes before and after treatments with anti-CaM (n = 10). B, Left, Noninvasive scanning ion-selective electrode test showed that 30 µM W7-agarose induced a rapid increase in extracellular Ca2+ influx and then it fluctuated between influx and efflux. Right, mean values for net Ca2+ influxes before and after treatments withW7-agarose (n = 7). (TIF) [file pone.0055411.s006.tif]

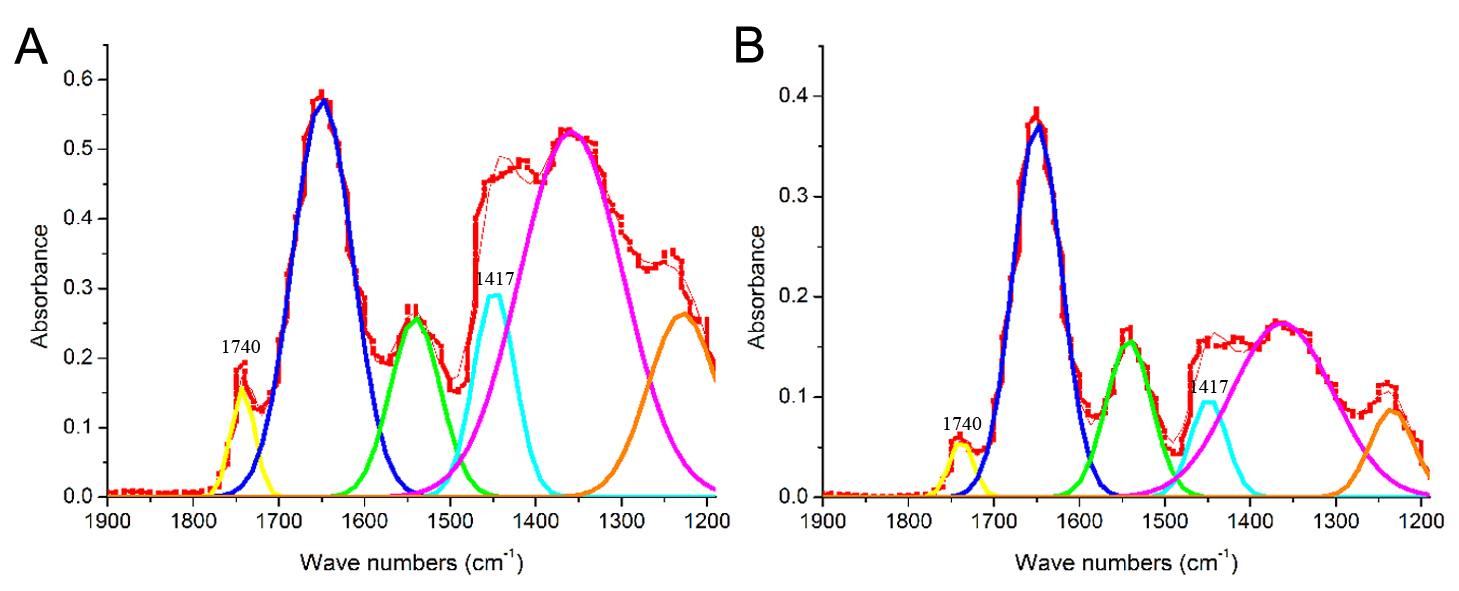

Supplement: Figure S7 — Gaussian fitting for FTIR spectra to quantify relative contents of esterified and acidic pectins. A, Gaussian fitting results for FTIR spectra for control cells (r2 = 0.98613). B, Gaussian fitting results for FTIR spectra for pollen tubes treated with 1.5 µg/mL anti-CaM (r2 = 0.98957). FTIR spectra were subjected to Gaussian fitting to quantify relative contents of acidic and esterified pectins using Origin 9.0 software (Origin Lab). Ratio between esterified pectins and acidic pectins was calculated from percentages of characteristic peak for esterified pectins and characteristic peak for acidic pectins. Ratio between acidic pectins (light blue peak) and esterified pectins (yellow peak) decreased from 3.86±0.77 (n = 5) to 2.59±0.43 (n = 7), indicating that acidic pectin deposition significantly decreased while esterified pectin increased upon anti-CaM treatment. Proportion of protein peaks (peaks at 1650 and 1550 cm−1) to total absorption peaks was also calculated, giving values of 0.49±0.12 (n = 5) for control cells and 0.35±0.19 (n = 7) for anti-CaM treated pollen tubes. (TIF) [file pone.0055411.s007.tif]
