# Supplementary material for: Inhibition of Apoplastic Calmodulin Impairs Calcium Homeostasis and Cell Wall Modeling during Cedrus deodara Pollen Tube Growth
Source: PLoS One. 2013 Feb 6;8(2):e55411. doi: 10.1371/journal.pone.0055411 (PMC3566176; doi:10.1371/journal.pone.0055411)
Supplement: Table S1 — Semi-quantitative analysis for the interested protein bands in SDS-PAGE gels. The results were obtained from three independent SDS-PAGE gels by semi-quantitative analysis for the gray scale values from the interested protein bands with Image J software. (DOC) [file pone.0055411.s008.doc]

**Table S1 Semi-quantitative analysis for the interested protein bands in SDS-PAGE gels**

| Protein identity | Mean intensity ± SD (gray scale value) | | | |
| --- | --- | --- | --- | --- |
|  | 0 μg/mL | 0.8 μg/mL | 1.0 μg/mL | 1.5 μg/mL |
| 17 KDa band | 139.37 ± 17.82 | 120.93 ± 8.77 | 88.12 ± 5.37 | 10.20 ± 0.59 |
| 21 KDa band | 147.91 ± 13.23 | 124.61 ± 6.44 | 90.57 ± 5.34 | 14.01 ± 1.76 |
| 25 KDa band | 46.37 ± 3.56 | 68.39 ± 2.31 | 52.84 ± 1.99 | 5.19 ± 1.09 |
| 26 KDa band | 47.04 ± 4.95 | 71.41 ± 5.73 | 63.01 ± 4.37 | 6.68 ± 1.87 |

The results were obtained from three independent SDS-PAGE gels by semi-quantitative analysis for the gray scale values from the interested protein bands with Image J software.
