# Supplementary material for: Inhibition of Apoplastic Calmodulin Impairs Calcium Homeostasis and Cell Wall Modeling during Cedrus deodara Pollen Tube Growth
Source: PLoS One. 2013 Feb 6;8(2):e55411. doi: 10.1371/journal.pone.0055411 (PMC3566176; doi:10.1371/journal.pone.0055411)
Supplement: File S1 — Supplemental materials and methods for recovery test in Figure 1 , calcium influx measurement and Fluo-3/AM staining. (DOC) [file pone.0055411.s009.doc]

Materials and Methods

Recovery test

The recovery tests in Figure 1 were carried out according to the procedures as followed: the pollen tubes incubated in the presence of 1 μg/mL anti-CaM for three days were collected to remove anti-CaM, afterwards the pollen tubes were further incubated in standard medium for another 2 days before statistical analysis; while the pollen tubes incubated in the presence of 40 μM W7-agarose for two days were collected to remove W7-agarose, afterwards the pollen tubes were further incubated in standard medium for another 1 day before statistical analysis. Therefore, pollen germination percentage and pollen tube growth were determined 5 days after incubation in the presence of anti-CaM and 3 days after incubation in the presence of W7-agarose.

Measurement of Extracellular Ca^2+^ Influx

Pollens were incubated in the standard medium for 54 h and then collected for the analysis. Net Ca^2+^ flux was measured in Xu Yue (Beijing) Science and Technology Co., Ltd. by using non-invasive scanning ion-selective electrode technique (SIET, BIO-IM, Younger USA LLC, Amherst, MA 01002, USA). The microelectrodes were backfilled with 100 mM CaCl_2_ to a length of approximately 1 cm from the tip, then were front-filled with approximately 25 μM liquid ion exchange (LIXs; Ca^2+^, 21048, Sigma-Aldrich, Louis, MO 63103, USA). Prior to fluxes measurement, the microelectrode was calibrated with different concentrations of Ca^2+^ buffer (0.05 mM and 0.01 mM). Only electrodes with Nernstian slope >50 mV/decade were used in our study. Ion flux was calculated by Fick’s law of diffusion: J =−D (dc/dx), where J is the ion flux in the x direction, dc is the ion concentration difference, dx is the distance of microelectrode movement between two points and D is the ion diffusion coefficient in a particular medium (the diffusion coefficient of Ca^2+^ is 2.86×10^−5^ cm^−2^ s^−1^ at 25 ℃). 5-9 pollen tubes were used for subsequent data analysis.

Fluo-3/AM staining of cytoplasmic calcium

Fluo-3/AM ester, a Ca^2+^-sensitive dye (Sigma), was added from a stock solution of 1 mM Fluo-3/AM in dimethylsulfoxide (DMSO). The final DMSO concentration in the incubation solution was approximately 1%. Pollen tubes were grown in culture medium supplemented with 1.5 µg/mL anti-CaM for 3 d, while the control pollen tubes were cultured in the presence of 1% DMSO, which did not affect pollen germination and tube growth. Pollen tubes cultured in both the treatment and the control were then loaded with 20 μM Fluo-3/AM ester at 4°C for 2 h in the dark. After the 2 h loading, the suspension was removed from the refrigerator, washed three times with standard medium, and then placed at room temperature in the dark for 1 h. Samples of treatments with anti-CaM and controls were collected, mounted, and photographed under a Zeiss LSM 510 Meta laser scanning confocal microscope (Zeiss Co., Germany) (excitation at 488 nm and emission at 515 nm).
